# Vaseline in Constipation

**Published:** 1904-12

**Authors:** 


### VASELINE IN CONSTIPATION.

Dr. W. D. Robinson in the treatment of constipation has found that the heavy petroleum oils act as excellent lubricators, and are not absorbed, being eliminated unchanged. In constipation, he usually administers ordinary white vaseline with a little water or orange-juice, with excellent results. He has had much success with this treatment in disease, as well as in health; particularly in the severe constipation that often accompanies typhoid fever. He thinks that it restricts bacterial growth, largely because it forms an oily coating over the contents of the entire digestive tract. He has also found that an antiseptic may be carried all the way through the digestive canal, when given in heavy petroleum oils, such as vaseline. He has administered salol in this way, dissolving it in vaseline by means of heat; and, on testing the stools, has found that the salol had been carried through the digestive tract. He thinks that at times this treatment has constituted a valuable means of controlling intestinal fermentation.—*Medical Review of Reviews.*

